# Supplementary material for: Applying species distribution models in public health research by predicting snakebite risk using venomous snakes’ habitat suitability as an indicating factor
Source: Sci Rep. 2020 Oct 22;10:18073. doi: 10.1038/s41598-020-74682-w (PMC7582189; doi:10.1038/s41598-020-74682-w)
Supplement: Supplementary file 1 — Supplementary Information. [file 41598_2020_74682_MOESM1_ESM.pdf]

Supplementary Information

Applying species distribution models in public health research by predicting snakebite risk using venomous snakes' habitat suitability as an indicating factor

M. Yousefi, A. Kafash, A. Khani, N. Nabati

Appendix S1. Papers and books examined in this study.

Ahmadzadeh, F., Flecks, M., Torki, F. & Böhme, W. A new species of angular-toed gecko, genus *Cyrtopodion* (Squamata: Gekkonidae), from southern Iran. *Zootaxa* **2924**, 22-32 (2011).

Akbarpour, M., Shafiei, S., Sehhatisabet, ME. & Damadi, E. A new species of frog-eyed gecko, genus *Teratoscincus* Strauch,1863 (Squamata: Sphaerodactylidae), from southeastern Iran. *Zool. Middle East* **63**, 296-302 (2017).

Fathinia, B., Karamiani, R., Darvishnia, H., Heidari, N. & Rastegar-Pouyani, N. A new species of 167 *Carinatogecko* (Sauria: Gekkonidae) from Ilam Province, western Iran. *Amphib. Reptile Conserv.* **5**, 61-74 (2011).

Fathinia, B., Rastegar-Pouyani, E. & Shafaeipour, A. A new species of *Eirenis* (Ophidia: Colubridae) from highland habitats in southern Iran. *Zool. Middle East* **65**, 319-329 (2019).

Fathinia, B., Rastegar-Pouyani, E., Rastegar-Pouyani, N. & Darvishnia, H. A new species of the genus *Rhynchocalamus* Günther, 1864 (Reptilia: Squamata: Colubridae) from Ilam Province in western Iran. *Zootaxa* **4282**, 473–486 (2017).

Fathinia, B., Rastegar-Pouyani, N. & Rastegar-Pouyani, E. Molecular phylogeny and historical biogeography of genera *Eristicophis* and *Pseudocerastes* (Ophidia, Viperidae). *Zool Scr.* **47**, 673–685 (2018).

Fathinia, B., Rastegar-Pouyani, N., Rastegar-Pouyani, E., Toodeh-Dehghan, F. & Rajabizadeh M. Molecular systematics of the genus *Pseudocerastes* (Ophidia: Viperidae) based on the mitochondrial cytochrome b gene. *Turk. J. Zool.* **38**, 575–581 (2014).

Gholamifard, A. *et al.* A new species of the genus *Microgecko Nikolsky*, 1907 (Sauria: Gekkonidae) from southern Iran. *Zootaxa* **4093**, 026-040 (2016).

Gholamifard, A., Rastegar-Pouyani, N. & Rastegar-Pouyani, E. A new species of the genus *Microgecko* Nikolsky, 1907 (Sauria: Gekkonidae) from the southern Zagros Mountains, Iran. *Zootaxa* **4648**, 435–454 (2019).

Hosseinian Yousefkhani, SS., Yousefi, M., Khani, A. & Rastegar Pouyani, E. Snake fauna of Shirahmad wildlife refuge and Parvand protected area, Khorasan Razavi province, Iran. *Herpetol. Notes* **7**, 75–82 (2014).

Kami, H.G. & Babaei Savasari, R. New records of the Turkmenian Fat-Tailed Gecko, *Eublepharis turcmenicus* Darevsky, 1978, from Khorasan Razavi Province, Iran (Squamata: Eublepharidae). IJAB **13**, 263–273 (2017).

Krause, V., Ahmadzadeh, F., Moazeni, M., Wagner, P. & Wilms, T.M. A new species of the genus *Tropiocolotes* Peters, 1880 from western Iran (Squamata: Sauria: Gekkonidae). *Zootaxa* **3716**, 22–038 (2013).

Latifi, M. *The Snakes of Iran* (Department of Environment, Tehran, 2000).

Moradi, N., Rastegra-Pouyani, N. & Rastegra-Pouyani, E. Geographic variation in the morphology of *Macrovipera lebetina* (Linnaeus, 1758) (Ophidia: Viperidae) in Iran. *Acta Herpetol.* **9**, 187–202 (2014).

Moradi, N., Shafiei, S. & Sehhatisabet, ME. The snake fauna of Khabr National Park, southeast of Iran. *IJAB* **9**, 41–55 (2013).

Mozafari, O., Kamali, K. & Fahimi H. *Atlas of Reptiles of Iran*. (Jahad Daneshgahi Press, Tehran, 2016).

Nasrabadi, R., Rastegar-Pouyani, E., Hosseinian Yousefkhani, S.S. & Khani, A. A checklist of herpetofauna from Sabzevar, Northeastern Iran. *Ira. J. Anim. Biosys*. **12**, 255-259 (2016).

Oraie H. *et al*. Molecular and morphological analyses have revealed a new species of blunt-nosed viper of the genus *Macrovipera* in Iran. *Salamandra* **54**, 233–238 (2018).

Rajabizadeh, M. *Snakes of Iran* (IranShenasi Publishing, Tehran, 2017).

Rounaghi, I., Rastegar-Pouyani, E. & Hosseinian, S. A new species of the genus *Tropiocolotes* peters, 1880 from Hormozgan Province, Southern Iran (Reptilia: Gekkonidae). *South-west. J. Hortic. Biol. Environ.* **9**, 15-23 (2018).

Sabbaghzadeh, A. & Mashayekhi, M. Survey of reptile fauna of Nazmabad of Arak, Markazi Province, Iran. *AJBAS* **7**, 101–108 (2015).

Sadeghi, R. & Torki, F. Notes on the natural history and distribution of *Carinatogecko stevenandersoni* Torki, 2011. *Amphib. Reptile Conserv*. **5**, 34-36 (2011).

Salemi, A., Heydari, N. & Jahan Mahin, M. A new distribution record for the rare Maynard's Longnose Sand Snake, *Lytorhynchus maynardi* Alcock and Finn, 1896 from Nikshahr, southeastern Iran. *Herpetol. Notes* **11**, 617–619 (2018).

Sami, S., Safaei-Mahroo, B. & Ghaffari, H. Range extensions of three endemic snake-skinks (Scincidae: *Ophiomorus*) in Iran. *Russ. J. Herpetol*. **24**, 329 – 332 (2017).

Torki, F. A new species of dwarf gecko of the genus *Microgecko* (Squamata: Gekkonidae) from Iran. *Sauria* **42**, 41–54 (2020).

Torki, F. Description of a new species of *Carinatogecko* (Squamata: Gekkonidae) from Iran. *Salamandra* **47**, 103–111 (2011).

Torki, F. Distribution, lifestyle, and behavioral aspects of the Iranian Fat-tailed Gecko, *Eublepharis angramainyu* Anderson and Leviton, 1966. *Gekkota* **6**, 17-22 (2010).

Torki, F. Three new species of *Hemidactylus* Oken, 1817 (Squamata, Gekkonidae) from Iran. *Amphib. Reptile Conserv*. **13**, 239–258 (2019).

Torki, F., Ahmadzadeh, F., Ilgaz, Ç., Avcı, A. & Kumlutaş, Y. Description of four new *Asaccus* Dixon and Anderson, 1973 (Reptilia: Phyllodactylidae) from Iran and Turkey. *Amphib. Reptil*. **32**, 185-202 (2011).

Yousefi, M., Khani, A., Shaykhi Ilanloo, S., Kafash, A. & Rastegar Pouyani, E. Reptile fauna of the Khajeh protected area, with assessing its similarities with physiogeographical area of the Iranian Lizards. *TBJ* **22**, 13–22 (2015).

Table S1. Provincial distribution of *Macrovipera lebetina*, *Echis carinatus*, *Naja oxiana and Pseudocerastes persicus* in Iran.

Number	Province	Species			
		Macrovipera lebetina	*Echis carinatus*	*Naja oxiana*	*Pseudocerastes persicus*
1	West Azerbaijan				
2	East Azerbaijan				
3	Ardabil				
4	Kurdistan				
5	Zanjan				
6	Gilan				
7	Kermanshah				
8	Hamedan				
9	Qazvin				
10	Alborz				
11	Mazandaran				
12	Ilam				
13	Lorestan				
14	Markazi				
15	Qom				
16	Tehran				
17	Khuzestan				
18	Chahar Mahaal and Bakhtiari				
19	Isfahan				
20	Semnan				
21	Golestan				

22	Khorasan-e-Shomali				
23	Khorasan-e-Razavi				
24	Yazd				
25	Khorasan-e-Jonobi				
26	Kohgiluyeh and Boyer-Ahmad				
27	Fars				
28	Kerman				
29	Sistan and Baluchestan				
30	Bushehr				
31	Hormozgan				
